# UNMET REHABILITATION NEEDS IN THE FIRST 6 MONTHS POST-INJURY IN A TRAUMA CENTRE POPULATION WITH MODERATE-TO-SEVERE TRAUMATIC INJURIES

**DOI:** 10.2340/jrm.v56.40078

**Published:** 2024-05-27

**Authors:** Håkon Øgreid MOKSNES, Nada ANDELIC, Christoph SCHÄFER, Audny ANKE, Helene Lundgaard SOBERG, Cecilie RØE, Emilie Isager HOWE, Marit V. FORSLUND, Olav RØISE, Hilde Margrete DAHL, Frank BECKER, Marianne LØVSTAD, Paul B. PERRIN, Juan LU, Unni SVEEN, Torgeir HELLSTRØM, Mari S. RASMUSSEN

**Affiliations:** 1 Department of Physical Medicine and Rehabilitation, Oslo University Hospital, Oslo; 2 Institute of Health and Society, Research Centre for Habilitation and Rehabilitation Models & Services (CHARM), Faculty of Medicine, University of Oslo, Oslo; 3 Department of Clinical Medicine, Faculty of Health Sciences, UiT the Arctic University of Norway, Tromsø; 4 Department of Rehabilitation, University Hospital of North Norway, Tromsø; 5 Faculty of Health Sciences, Oslo Metropolitan University, Oslo; 6 Institute of Clinical Medicine, Faculty of Medicine, University of Oslo, Oslo; 7 Norwegian Trauma Registry, Division of Orthopaedic Surgery, Oslo University Hospital, Oslo; 8 Department of Clinical Neurosciences for Children, Oslo University Hospital, Oslo; 9 Department of Research, Sunnaas Rehabilitation Hospital, Bjørnemyr; 10 Department of Psychology, Faculty of Social Sciences, University of Oslo, Oslo, Norway; 11 School of Data Science, University of Virginia, Charlottesville, VA; 12 Department of Psychology, University of Virginia, Charlottesville, VA; 13 Central Virginia Veterans Affairs Health Care System, Richmond, VA; 14 Department of Epidemiology, School of Population Health, Virginia Commonwealth University, Richmond, USA

**Keywords:** assessment of healthcare needs, community health services, rehabilitation, multiple trauma, traumatic injury, trauma centre

## Abstract

**Objective:**

To describe the needs for subacute inpatient rehabilitation and community-based healthcare services, rehabilitation, and social support in patients with moderate-to-severe traumatic injury in the first 6 months post-injury. Further, to explore associations between sociodemographic and clinical characteristics and unmet needs.

**Design:**

Multicentre prospective cohort study.

**Subjects:**

Of 601 persons (75% males), mean (standard deviation) age 47 (21) years, admitted to trauma centres in 2020 with moderate-to-severe injury, 501 patients responded at the 6-month follow-up and thus were included in the analyses.

**Methods:**

Sociodemographic and injury-related characteristics were recorded at inclusion. Estimation of needs was assessed with the Rehabilitation Complexity Scale Extended–Trauma and the Needs and Provision Complexity Scale on hospital discharge. Provision of services was recorded 6 months post-injury. Multivariable logistic regressions explored associations between baseline variables and unmet inpatient rehabilitation and community-based service needs.

**Results:**

In total, 20% exhibited unmet needs for subacute inpatient rehabilitation, compared with 60% for community-based services. Predictors for unmet community-based service needs included residing in less central areas, profound injury severity, severe head injury, and rehabilitation referral before returning home.

**Conclusion:**

Inadequate provision of healthcare and rehabilitation services, particularly in the municipalities, resulted in substantial unmet needs in the first 6 months following injury.

LAY ABSTRACT

Injuries are a common cause of disability. However, it is unclear if patients’ needs for rehabilitation after injury are met with sufficient services. In this study, we followed 601 patients for the first 6 months after moderate or severe physical injury. Patients’ needs for health and rehabilitation services post-injury were predicted upon discharge from the acute hospital. At the 6-month follow-up, we asked patients what health and rehabilitation services they had received. Results indicated that, for 1 in 5, needs for rehabilitation in hospital or specialized institution were unmet, and 6 out of 10 had unmet needs for community-based health and rehabilitation services and social support. Risk factors for unmet needs 6 months post-injury included having a severe injury, especially a severe head injury, and living in less central regions. A need for improvement of services exists, especially for patients living outside central areas and those with severe injuries.

Physical injuries sustained to an individual’s extremities, head, chest, or spine are among the leading causes of physical, cognitive, emotional, and behavioural impairments and vocational disability (1–3). While improved quality of acute trauma care has reduced mortality (4, 5), a substantial proportion of individuals who experience moderate or severe trauma have residual functional impairments requiring complex short- and long-term rehabilitation efforts (2, 6–8).

Previous research suggests that rehabilitation after trauma improves patients’ functional outcomes beyond those expected from spontaneous recovery (9, 10). Such rehabilitation requires comprehensive care and services provided by medical, allied health, and social care professionals. A *need for rehabilitation* refers to any need that can be met with inpatient and outpatient services as well as community-based services in the acute and post-acute phases of an injury (11). A discrepancy between rehabilitation provision and the need for healthcare and rehabilitation services has been documented internationally (11). In a recent Australian pilot study, 87% of trauma survivors not receiving inpatient rehabilitation had unmet rehabilitation needs following hospital discharge (12). These findings are in line with a large European study on traumatic brain injury (TBI) that reported almost 90% of patients with moderate-to-severe disability within 6 months of injury had unmet rehabilitation needs in several functional domains (13).

Healthcare and rehabilitation service use varies based on demand, sociodemographic, and injury characteristics. Service provision and access further vary across regions and countries. Inconsistencies exist in international research regarding regional disparities between high- and low-density population areas (11). While geographical variations in access to rehabilitation services have been demonstrated in Australia (14), a cohort study from multiple trauma survivors in Canada found no regional differences in rehabilitation services although numerous barriers to rehabilitation were reported (15). Older age, female sex, and severe trauma are associated with worse outcomes, and these patients require additional support and rehabilitation services (3, 16). Further, Andelic et al. reported that unmet rehabilitation needs were significantly higher in individuals with a less severe disability after TBI (17). Thus, more research on regional variations, clinical characteristics, recovery, and rehabilitation needs is warranted to optimize acute and post-acute services after traumatic injuries (14).

New knowledge concerning rehabilitation needs will improve our understanding of current service provision and the gaps between needs and services, which can be used to improve rehabilitation and healthcare resource planning and allocation. The primary aim of the present study was to describe the needs for subacute inpatient rehabilitation and community-based healthcare and rehabilitation services among patients with traumatic injuries within the first 6 months post-injury. A secondary aim was to explore the association among sociodemographic factors, clinical characteristics, and unmet healthcare and rehabilitation needs. We anticipated that the need to be transferred to and receive subacute inpatient rehabilitation would largely be met, whereas unmet needs for community-based healthcare and rehabilitation services would be high in the first 6 months post-injury.

## METHODS

### Participants and participant recruitment

This prospective cohort study included patients of all ages with moderate-to-severe traumatic injury admitted to the regional trauma centres at Oslo University Hospital (OUH) and the University Hospital of North Norway Tromsø (UNN). The study protocol (18) and epidemiological characteristics detailing inclusion and recruitment procedures have been published elsewhere (19). A consecutive cohort was recruited from January 2020 to December 2020 (OUH) and February 2020 to January 2021 (UNN). Patients were enrolled during their stay at the trauma care unit or immediately after discharge. The baseline for all patients was the date of trauma. Patient follow-ups were performed at 6 and 12 months post-injury. In this study, we analysed data gathered at the 6-month follow-up.

### Inclusion and exclusion criteria

Patients were eligible for inclusion if they had sustained a moderate-to-severe traumatic injury, defined as New Injury Severity Score (NISS) > 9 (2008 update of the 2005 Abbreviated Injury Scale [AIS]) (20). Other inclusion criteria were admission to the trauma centre within 72 hours after the injury and length of hospital stay ≥ 2 days. Patients who were non-Norwegian residents, had insufficient understanding of Norwegian or English languages, or died before discharge were excluded.

### Data collection

Data collection methods, including the recruitment of patients, have been described in detail elsewhere (19).

### Patient characteristics

*Sociodemographic variables.* Sociodemographic variables included age, sex, pre-injury comorbidity assessed using the American Society of Anesthesiologists Physical Status Classification System (ASA-PS) (21), and pre-injury mental health or drug/alcohol condition. The latter was assigned if information regarding the condition was found in the medical record. ASA-PS scores were dichotomized into healthy (ASA-PS score 1) and systemic disease (ASA-PS score 2–4, none assigned score 5 or 6). Geographical centrality was classified according to the Norwegian Centrality Index (NCI) defined by the time of travel to workplaces and other official services and applied to the patients’ municipality of residency (22). The NCI was dichotomized into central (NCI 1–2) and less central (NCI 3–6).

*Clinical variables.* The number of injuries and AIS and NISS scores were collected from the trauma registers of the 2 hospitals, both of which collect data for the Norwegian Trauma Registry. NISS scores were categorized as follows: moderate = 10–15, severe = 16–24, and profound > 24. An AIS score ≥ 3 was considered a severe injury, and AIS (body region) was dichotomized into less severe (AIS < 3) vs severe (AIS ≥ 3). Length of hospital stay (LOS) was defined as the number of days in the acute care units at the trauma centres. Discharge destinations were dichotomized as discharged to home/local hospitals vs specialized rehabilitation (defined as rehabilitation in a hospital or institution that is a part of the specialist healthcare system).

### Main outcomes

Unmet rehabilitation needs were assessed as the discrepancies between the estimated needs at the time of discharge from the trauma departments and the services provided in the 6 first months after the injury as reported by the patients and/or caregivers. The estimated needs on discharge were based on the Rehabilitation Complexity Scale Extended–Trauma (RCS E–Trauma) and the Needs and Provision Complexity Scale (NPCS).

### Rehabilitation Complexity Scale Extended–Trauma (RCS E - Trauma)

The needs for subacute inpatient rehabilitation in the specialist healthcare system during the first 6 months post-injury were estimated using the RCS E–Trauma, which reflects rehabilitation resource requirements. It consists of 7 domains and 5 subscales. The domains are: medical needs (M, 0–6), basic care and support needs (C, 0–4) and risk (cognitive or behavioural needs) (R, 0–4), skilled nursing needs (N, 0–4), number of different therapy disciplines required (TD, 0–4) and therapy intensity (TI, 0–4), and equipment needs (E, 0–3). The RCS E–Trauma sum score is computed as the sum of the subscale scores M, C/R (the largest of scores C and R), N, TD + TI, and E, providing a sum score range of 0–25 (23, 24).

Subacute in-hospital rehabilitation was defined as rehabilitation commencing after acute treatment at the specialist care level and provided by either 1 (general rehabilitation) or more therapeutic professionals/allied health professionals (multidisciplinary rehabilitation) in addition to physicians and nurses. The needs for the first 6 months were estimated by the RCS E–Trauma per clinical and evidence-based judgement by doctors specialized in rehabilitation medicine (authors HM, CS, and NA) on discharge from acute care at the trauma hospitals. The inter-rater reliability (subscale scores) among the 3 rehabilitation specialists were calculated with intra-class correlations (ICC). The calculations were based on a sample of 11 patients and were excellent for consistency (ICC 0.959) and good for absolute agreement (ICC 0.899) (25). For the evaluation of met subacute rehabilitation needs during the 6 months, the doctors used information from medical records (rehabilitation discharge reports if available) and the telephone interview with patients at the 6-month follow-up to evaluate whether estimated needs were met.

### Needs and Provision Complexity Scale (NPCS)

The NPCS is a 15-item measure used to evaluate met and unmet needs for community-based healthcare services and social care/rehabilitation and support (26). The NPCS clinician version assesses an individual’s needs for rehabilitation and support (NPCS-Needs) within a given period. The level of services provided (NPCS-Gets), is obtained from the patient version of the NPCS. The 15 items are divided into 2 principal domains: health and personal care (0–25) and social care and support (0–25). Health and personal care needs include the following subscales: healthcare (score range 0–6), personal care (score range 0–10), and rehabilitation (score range 0–9). The social care and support needs domain includes the following subscales: social and family support (score range 0–13) and environment (score range 0–12) (26). The total scale ranges from 0–50 with higher scores indicating more needs. The discrepancy score between NPCS-Needs and NPCS-Gets displays the level of unmet needs. In the present study, we used the clinician NPCS-Needs completed at the time of discharge from the trauma centre. The patient NPCS-Gets was completed by the telephone interviewer at the 6-month follow-up. The estimated needs for community-based services in the first 6 months were estimated per clinical and evidence-based judgement by doctors specialized in rehabilitation medicine (authors HM, CS, and NA) on discharge from acute care at the trauma hospitals. The inter-rater reliability (subscale scores) among the 3 rehabilitation specialists calculated with intra-class correlations (ICC) was good for both consistency (ICC 0.858) and absolute agreement (ICC 0.756) (25). The calculation was based on a sample of 11 patients.

### Statistical analyses

Descriptive analyses were performed to summarize and present patients’ characteristics. The level of unmet needs is presented as the discrepancies between the estimates and the provision of services as registered in the RCS E–Trauma and the NPCS. To facilitate comparison, we used the same scoring method on both scales. Met and unmet needs were calculated for total, domain, and corresponding subscale scores. Levels of unmet needs were calculated for the total sample and 3 different injury severity groups: moderate (NISS = 10–15), severe (NISS = 16–24), and profound (NISS > 24).

Multiple logistic regression analyses were conducted to identify factors predicting unmet needs in the first 6 months after injury. The total scores of the RCS E–Trauma and the NPCS were dichotomized into met needs (≤ 0, including exceeded needs) and unmet needs (> 0). The total scores of the scales were applied as dependent variables in the model as well as the scores of the health and personal care and the social care and support domains of the NPCS (see Tables SI–SIII).

As per Wald statistics, factors with *p*-values below the recommended removal criterion of 0.25 in simple logistic regression analyses were included in the multivariate logistic regression models. Further, factors considered relevant based on previous research and clinical experience were also included in the models to investigate their associations with unmet needs. We applied similar multiple regression models to both instruments to demonstrate that certain factors are common and consistently important. To control for the heterogeneity in the included sample, all models were adjusted for age (years), sex (male vs female), geographical region (central/less central), comorbidity as assessed with ASA (no/yes), number of injuries, and severity of injured body regions based on AIS scores ≥ 3. Results from the multiple logistic regression analyses are presented with odds ratios (ORs) with 95% confidence intervals. Furthermore, we computed Hosmer–Lemeshow goodness-of-fit, -2 Log likelihood, Cox and Snell R^2^, and Nagelkerke R^2^ statistics. Before conducting the logistic regressions, we investigated multicollinearity. Variables with high correlation coefficients (≥ 0.70) were not entered into the regression analyses. The significance level was set to *p* < 0.05. All analyses were conducted using SPSS version 28.0 (IBM Corp, Armonk, NY, USA).

## RESULTS

**Fig. 1:**
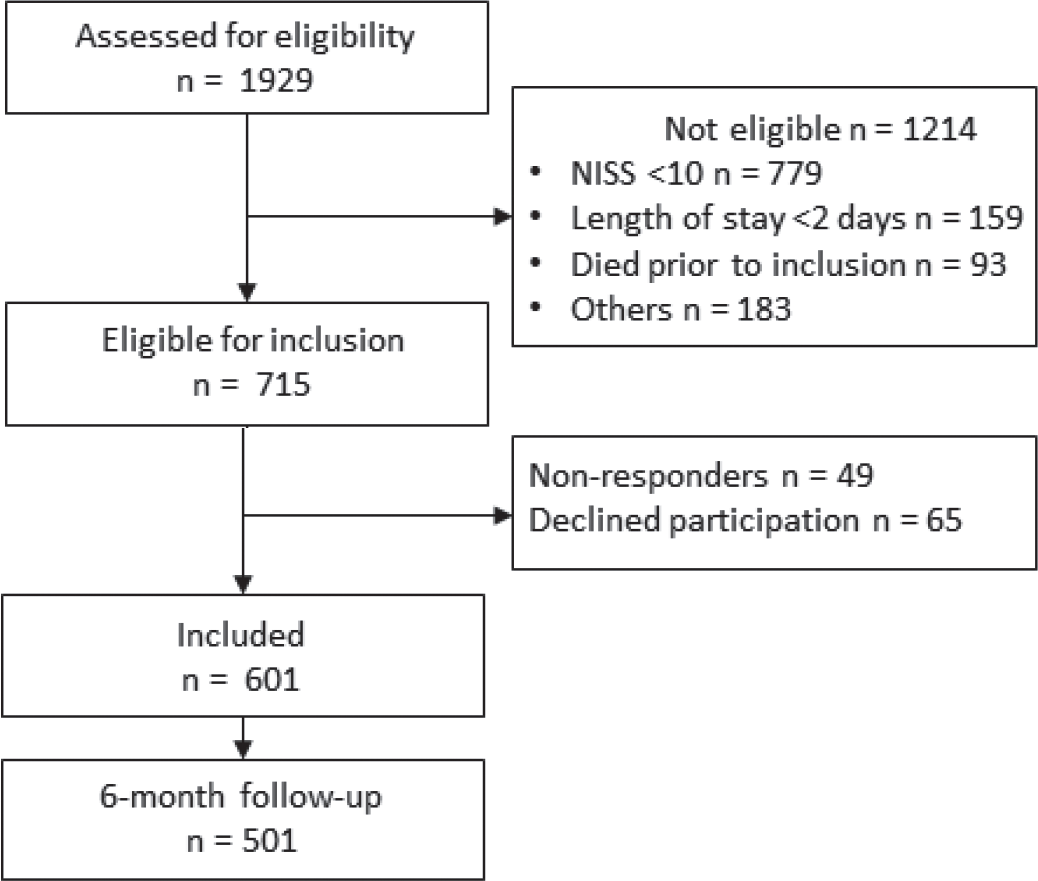
Flowchart. NISS: New Injury Severity Score.

The sociodemographic and clinical characteristics of the total sample and across injury severity groups are summarized in Table I. In total, 1,929 patients were assessed for eligibility, of whom 601 patients were included. Details of recruitment and study flow are presented in Fig. 1.

**Table I:** Sociodemographic and clinical characteristics of the total sample and across injury severity groups (*N* = 601)

Variable	Total (all participants)	Moderate severity NISS 10–15 (*n* = 144)	Severe NISS 16–24 (*n* = 211)	Profound severity NISS > 24 (*n* = 246)
Sex, *n* (%)				
Male Female	451 (75.0) 150 (25.0)	107 (74.3) 37 (25.7)	155 (73.5) 56 (26.5)	189 (76.8) 57 (23.2)
Age, years, mean (SD)	46.9 (21.2)	45.5 (22.0)	46.4 (21.3)	48.0 (20.6)
Living status, *n* (%)				
Living alone Living with others (partner/parents)	215 (35.9) 384 (64.1)	55 (38.5) 88 (61.5)	76 (36.2) 134 (63.8)	84 (34.1) 162 (65.9)
Geographical region, *n* (%)				
Central (Norwegian centrality index 1–2) Less central (Norwegian centrality index 3–6)	337 (56.1) 264 (43.9)	97 (67.4) 47 (32.6)	120 (56.9) 91 (43.1)	120 (48.8) 126 (51.2)
Pre-injury comorbidity (ASA), *n* (%)				
Healthy (ASA 0) Comorbidity (ASA 1–6)	327 (54.4) 274 (45.6)	90 (62.5) 54 (37.5)	116 (55.0) 95 (45.0)	121 (49.2) 125 (50.8)
Pre-injury mental health or drug/alcohol condition, *n* (%)				
Yes No	133 (23.7) 428 (76.3)	29 (22.0) 103 (78.0)	38 (19.2) 160 (80.8)	66 (28.6) 165 (71.4)
Injury mechanism, *n* (%)				
Falls Transport-related Sport accident/others Violence	243 (40.4) 227 (37.8) 113 (18.8) 18 (3.0)	52 (36.1) 55 (38.2) 33 (22.9) 4 (2.7)	76 (36.0) 79 (37.4) 49 (23.2) 7 (3.3)	115 (46.8) 93 (37.8) 31 (12.6) 7 (2.9)
Number of injuries, mean (SD)	6.1 (3.8)	3.8 (2.1)	5.3 (2.7)	8.1 (4.4)
Median [min, max]	5 [1, 26]	3 [2, 13]	5 [1, 18]	7 [1, 26]
Length of hospital stay, days, mean (SD)	8.6 (9.0)	6.1 (6.3)	7.2 (7.0)	11.2 (11.0)
Median [min, max]	6 [2, 104]	4 [2, 58]	5 [1, 51]	8 [1, 104]
Discharge destination, *n* (%)				
Local hospital/home, n (%) Specialized rehabilitation, *n* (%)	467 (77.7) 134 (22.3)	132 (91.7) 12 (8.3)	177 (83.9) 34 (16.1)	158 (64.2) 88 (35.8)

SD: standard deviation: ASA: American Society of Anesthesiologists classification; NISS: New Injury Severity Score.

In total, 501 patients (83.4%) responded at the 6-month follow-up and thus were included in the analyses. The mean age (SD) at the time of injury was 46.9 (21.2) years, 75% were male, and 56.1% lived in central geographical regions. According to the ASA score, approximately half (54%) were considered healthy. Approximately one-quarter (24%) had a pre-injury mental health or drug/alcohol condition.

The most common cause of injury was falls, followed by transport-related accidents. Regarding injury severity assessed by the NISS, most patients (76%) had severe injuries, 41% were classified as profound. Mean LOS was 8.6 days. In total, 77.7% of the patients were discharged home or to local hospitals from the acute care units at the trauma centres. Direct discharge to specialized rehabilitation was most common among those with profound injuries (35.8%).

### Needs for subacute inpatient rehabilitation and community-based healthcare, social support, and rehabilitation services (RCS E–Trauma and NPCS)

Frequencies and percentages of patients with unmet needs in the total sample and across injury severity groups are presented in Tables II and III.

**Table II:** Met and unmet needs for inpatient primary rehabilitation in the first 6 months post-injury for the total sample and across injury severity groups

RCS E–Trauma discrepancy 6-months follow-up	Unmet needs	All participants, *n* (%)	Moderate severity NISS 10–15 (*n* = 144) *n* (%)	Severe NISS 16–24 (*n* = 211) *n* (%)	Profound severity NISS > 24 (*n* = 246) *n* (%)
Total score (n = 498)	Met Unmet	395 (79.3) 103 (20.7)	90 (75.6) 29 (24.4)	138 (80.2) 34 (19.8)	167 (80.7) 40 (19.3)
Medical needs	Met Unmet	469 (94.2) 29 (5.8)	108 (90.8) 11 (9.2)	161 (93.6) 11 (6.4)	200 (96.6) 7 (3.4)
Basic care & support needs (including risk)	Met Unmet	472 (94.8) 26 (5.2)	111 (93.3) 8 (6.7)	164 (95.3) 8 (4.7)	197 (95.2) 10 (4.8)
Skilled nursing needs	Met Unmet	449 (90.2) 49 (9.8)	107 (89.9) 12 (10.1)	155 (90.1) 17 (9.9)	187 (90.3) 20 (9.7)
Therapy needs (disciplines and intensity)	Met Unmet	395 (79.3) 103 (20.7)	93 (78.2) 26 (21.8)	136 (79.1) 36 (20.9)	166 (80.2) 41 (19.8)
Equipment needs	Met Unmet	470 (94.4) 28 (5.6)	110 (92.4) 9 (7.6)	164 (95.3) 8 (4.7)	196 (94.7) 11 (5.3)

NISS: New Injury Severity Score; RCS E–Trauma: Rehabilitation Complexity Scale Extended–Trauma.

**Table III:** Met and unmet needs for community-based healthcare services and social care/rehabilitation for the total sample and across injury severity groups

NPCS 6-months follow-up	Unmet needs	All participants, *n* (%)	Moderate severity NISS 10–15 (*n* = 144) *n* (%)	Severe NISS 16–24 (*n* = 211) *n* (%)	Profound severity NISS > 24 (*n* = 246) *n* (%)
NPCS unmet needs total score (*n* = 501)	Met Unmet	204 (40.7) 297 (59.3)	62 (51.2) 59 (48.8)	78 (45.3) 94 (54.7)	64 (30.8) 144 (69.2)
Healthcare	Met Unmet	390 (77.8) 111 (22.2)	90 (74.4) 31 (25.6)	138 (80.2) 34 (19.8)	162 (77.9) 46 (22.1)
Personal care	Met Unmet	403 (80.4) 98 (19.6)	108 (89.3) 13 (10.7)	146 (84.9) 26 (15.1)	149 (71.6) 59 (28.4)
Rehabilitation	Met Unmet	242 (48.3) 259 (51.7)	67 (55.4) 54 (44.6)	98 (57.0) 74 (43.0)	77 (37.0) 131 (63.0)
Social and family support	Met Unmet	250 (49.9) 251 (50.1)	73 (60.3) 48 (39.7)	97 (56.4) 75 (43.6)	80 (38.5) 128 (61.5)
Environment	Met Unmet	440 (88.0) 60 (12.0)	106 (88.3) 14 (11.7)	151 (87.8) 21 (12.2)	183 (88.0) 25 (12.0)

NISS: New Injury Severity Score; NPCS: Needs and Provision Complexity Scale.

The mean (SD) and median (IQR) of estimated RCS E–Trauma total scores on discharge from the trauma centre were 8.2 (5.7) and 9 (1–12), respectively, whereas the respective values for received services at 6 month follow-up were 7.5 (5.6) and 8 (1–12). At baseline, 24% of patients had estimated needs corresponding to a total score of ≥ 12, indicating needs for complex rehabilitation services. Overall, 20.4% of participants had unmet needs on RCS E–Trauma assessed by the total score during the first 6 months post-injury. The proportion of patients with unmet subacute rehabilitation needs was comparably distributed across injury severity levels. Regarding the RCS E–Trauma domains/subscales, unmet needs for therapy disciplines and intensity of rehabilitation were the most frequently reported (approximately 20%), whereas needs in the other domains were met for the majority of patients. Of those with unmet needs, 5.6% received general rehabilitation (one therapist plus physician and nurse) and 14.8% of patients received multidisciplinary rehabilitation (mainly 2, 3, or 4 therapists plus physician and nurse).

**Fig. 2:**
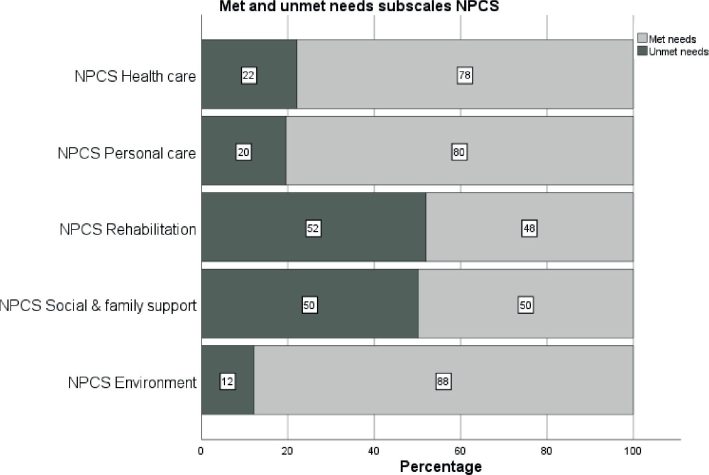
Bar chart illustrating the proportions of met and unmet needs on the different subscales of the Needs and Provision Complexity Scale (NPCS).

The estimated mean (SD) and median (IQR) NPCS total scores on discharge from the trauma centre were 10.3 (6.0) and 10 (6–13), respectively, whereas mean (SD) and median (IQR) values for received services at 6-month follow-up were 5.9 (5.9) and 5 (2–8), respectively. The highest estimated needs were within the rehabilitation subscale with a mean of 3.4 (1.9) followed by the healthcare subscale with a mean of 2.7 (1.2). Almost 60% of the patients had unmet community-based service needs based on the total score of the NPCS during the first 6 months after injury. The highest proportion of patients with unmet needs was among those with profound injuries (NISS > 24), of whom 69% had unmet needs. The most frequently unmet needs were within the subscales of rehabilitation (including therapy disciplines/intensity and vocational/educational support) and social and family support (52% and 50%, respectively), with patients with profound injuries showing higher frequencies (63% and 62%, respectively). Conversely, the most frequently met needs were from the personal care and environment subscales (Fig. 2).

### Factors associated with levels of unmet needs

The results of modelling of unmet needs (RCS E–Trauma and NPCS) total scores are presented in Tables IV and V, respectively.

**Table IV:** Sociodemographic and clinical factors associated with unmet needs on the Rehabilitation Complexity Scale Extended–Trauma at 6 months post-injury in multiple logistic regression analysis (*n* = 465)

Variables	OR (95% CI)	*p*-value
Sex, female vs male^a^	1.548 (0.901–2.661)	0.114
Age (years)	0.996 (0.982–1.010)	0.594
Living with others vs living alone^a^	1.384 (0.794–2.415)	0.252
Less central (NCI = 3–6) vs central (NCI = 1–2) geographical region^a^	1.547 (0.943–2.536)	0.084
Pre-injury comorbidity, systemic disease (ASA = 2–4) vs healthy (ASA = 1)^a^	1.811 (0.981–3.342)	0.058
Pre-injury mental health or drug/alcohol condition, no vs yes^a^	0.850 (0.422–1.711)	0.648
Overall injury severity, NISS ≥ 26 vs NISS ≤ 25^a^	1.233 (0.655–2.323)	0.516
Number of injuries	0.986 (0.911–1.066)	0.715
Head injury severity, AIS ≥ 3 vs < 3^a^	1.183 (0.609–2.297)	0.620
Thorax injury severity, AIS ≥ 3 vs < 3^a^	0.719 (0.389–1.329)	0.293
Abdomen injury severity, AIS ≥ 3 vs < 3^a^	0.215 (0.070–0.661)	0.007*
Spine injury severity, AIS≥ 3 vs < 3^a^	1.556 (0.788–3.074)	0.203
Lower extremities injury severity, AIS ≥ 3 vs < 3^a^	0.842 (0.417–1.700)	0.632
Discharge destination, specialized rehabilitation vs local hospital/home/other^a^	0.218 (0.101–0.470)	< 0.001*

a Reference group; OR > 1 increases the odds of having a high level of unmet needs; OR < 1 decreases the odds of having a high level of unmet needs. Hosmer and Lemeshow goodness-of-fit test χ^2^ = 9.560, df = 8, *p* = 0.297; -2 log likelihood = 422,306; Cox & Snell R^2^ = 0.083; Nagelkerke R^2^ = 0.132.

AIS: Abbreviated Injury Scale; ASA: American Society of Anesthesiologists classification; CI: confidence interval; NCI: Norwegian Centrality Index; NISS: New Injury Severity Score; OR: odds ratio; RCS E–Trauma: Rehabilitation Complexity Scale Extended–Trauma.

* *p*-value < 0.05.

**Table V:** Sociodemographic and clinical factors associated with unmet community-based healthcare needs on Needs and Provision Complexity Scale during the first 6 months post-injury in multiple logistic regression analysis (*n* = 465)

Variables	OR (95% CI)	*p*-value
Sex, female vs male^a^	1.313 (0.823–2.096)	0.253
Age (years)	1.004 (0.993–1.016)	0.474
Living with others vs living alone^a^	0.899 (0.580–1.395)	0.635
Less central (NCI = 3–6) vs central (NCI = 1–2) geographical region^a^	1.885 (1.250–2.842)	0.002*
Pre-injury comorbidity (ASA) vs healthy^a^	1.099 (0.667–1.809)	0.266
Pre-injury mental health or drug/alcohol condition, no vs yes^a^	0.858 (0.491–1.499)	0.590
Number of injuries	0.965 (0.906–1.028)	0.264
NISS ≥ 26 vs NISS ≤ 25^a^	1.833 (1.083–3.101)	0.024*
Head AIS ≥ 3 vs < 3^a^	1.745 (1.012–3.009)	0.045*
Thorax AIS ≥ 3 vs < 3^a^	0.690 (0.418–1.141)	0.148
Abdomen AIS ≥ 3 vs < 3^a^	0.808 (0.428–1.526)	0.512
Spine AIS ≥ 3 vs < 3^a^	0.936 (0.437–1.413)	0.421
Lower extremities AIS ≥ 3 vs < 3^a^	1.718 (0.951–3.103)	0.074
Discharge destination specialized rehabilitation vs home/local hospital/others^a^	1.757 (1.011–3.052)	0.046*

a Reference group; OR > 1 increases the odds of having unmet needs; OR < 1 decreases the odds of having unmet needs. Hosmer and Lemeshow goodness-of-fit test χ^2^ (8) = 21,138, *p* = 0.07; -2 log likelihood = 580,230; Cox & Snell R^2^ = 0.110; Nagelkerke R^2^ = 0.148.

AIS: Abbreviated Injury Scale; ASA: American Society of Anesthesiologists classification; CI: confidence interval; NCI: Norwegian Centrality Index; NISS: New Injury Severity Score; OR: odds ratio.

* *p*-value < 0.05.

Results from the logistic regression model including RCS E–Trauma (total sum) (Table IV) showed that living in less central regions and having pre-injury comorbidity (ASA) increased the odds of having unmet needs for subacute rehabilitation by 1.6 and 1.8 times, respectively. Having a severe abdominal injury (AIS ≥ 3) or being discharged directly to specialized rehabilitation decreased the odds of having unmet needs by 0.8 times. We did not perform logistic regression analyses on the RCS E–Trauma subdomains due to the limited number of patients with unmet needs.

The results from the NPCS multiple logistic regression analyses (Table V) demonstrated that living in less central areas increased the odds of unmet needs for community-based healthcare and rehabilitation services by 1.9 times. Having an injury classified as profound on the NISS increased the odds of unmet needs by 1.8 times, and having a head injury (AIS ≥ 3) increased the odds by 1.8 times. Being discharged directly to specialized rehabilitation increased the odds of having unmet needs by 1.8 times compared with being discharged to home/local hospitals.

When modelling unmet needs for the NPCS principal domain of health and personal care, we found that patients living in less central areas (OR 1.77), having a higher head AIS score (OR 2.53), or being discharged to specialized rehabilitation rather than home/local hospitals (OR 1.79) were more likely to experience unmet needs (Table SI).

Living in less central areas comparably increased the odds of reporting unmet needs (OR 2.14) in the domain of social care and support. Additionally, having a more severe spinal injury (OR 1.81), lower extremity AIS ≥ 3 (OR 2.92), and being discharged to specialized rehabilitation (OR 2.31) increased the odds of reporting unmet needs. Not having a pre-injury mental health or drug/alcohol condition decreased the odds of unmet needs (OR 0.46) (Table SII).

A similar pattern was observed for living in less central areas (OR 2.07) and head AIS ≥ 3 (OR 2.56) when modelling the NPCS rehabilitation subscale. In this model, we also found that profound injuries increased the odds of unmet needs (OR 1.67), whereas having fewer injuries decreased the odds of having unmet rehabilitation needs (OR 0.93) (Table SIII).

## DISCUSSION

This study describes the level of met and unmet needs and their associative factors for both subacute inpatient rehabilitation and community-based healthcare and rehabilitation in a trauma centre population cohort within the first 6 months after moderate-to-severe trauma. Our results demonstrate that needs for subacute inpatient rehabilitation were mostly met, but still 1 in 5 had unmet needs. Moreover, more than half of patients had unmet needs for community-based health services, social support, and rehabilitation services (and the highest proportion of unmet needs were within the subscale rehabilitation of the NPCS).

We used the RCS E–Trauma to estimate needs for subacute general and specialized inpatient rehabilitation on discharge from the trauma centres. Given the shortage of similar studies among trauma populations, few comparisons are possible. However, RCS E–Trauma data exist in the National Clinical Audit of Specialist Rehabilitation following major Injury in the UK (NCASRI) (27). About 24% of patients in our study had estimated complex service needs (total RCS E–Trauma score of 12 and above, and may correspond to Category A/B or tertiary and local specialist rehabilitation services in the NCASRI system), in contrast to 55% of patients in the NCASRI project. This may indicate different study inclusion criteria, differences between health systems and rehabilitation organizations, or more severe traumatic injuries in the UK compared with Norway. Further, a potential underestimation of complex needs for subacute inpatient rehabilitation services cannot be ruled out. Three experienced rehabilitation specialists estimated these needs on discharge from acute stay at the trauma centres, and the estimations could have been biased by the outlook and experience of the specialist and the available services in Norway. However, inter-rater reliability among the specialists was good to excellent, which strengthens an assumption that the estimates were legitimately precise.

At the 6-month follow-up, we found that most patients’ subacute inpatient rehabilitation services needs had been met. However, given the substantial proportion of patients without estimated needs for inpatient rehabilitation, 20% having unmet primary rehabilitation needs among all patients with moderate-to-severe injuries appears significant. A previous study on TBI found that the odds of receiving rehabilitation services are higher for patients living in Northern Europe, probably due to the high numbers of rehabilitation professionals and the public welfare systems (28). In the present study, the only injury severity-related factor found to be predictive of lower unmet needs for these services was abdominal injury severity AIS ≥ 3 vs < 3. Many patients with injuries to the abdominal organs without additional injuries will be treated without surgery and then discharged home. Even those who receive surgical treatment (i.e. laparotomy) without sustaining other severe injuries are often discharged home when their bowel function is re-established. Of the clinical/treatment-related factors, being discharged to subacute rehabilitation was associated with lower unmet rehabilitation service needs. This is in line with patients’ reports in clinical follow-up settings, at least at the OUH, where they rate the needs for subacute inpatient rehabilitation as mainly covered in contrast to rehabilitation services in the municipalities (personal communication).

### Needs and Provision Complexity Scale (NPCS)

As hypothesized, we found a high proportion of patients (60%) with unmet needs in provision of health-care and social support services in the community across all injury severity levels. The NPCS has previously been used in long-term neurological conditions as an assessment of unmet needs (26, 29, 30) and was recently validated in Norway for TBI and subarachnoid haemorrhage (31). Siegert et al. identified significant gaps in service provision compared with patients’ needs within the first year following discharge from inpatient neurorehabilitation, especially relating to rehabilitation services, social and family support, and equipment (29). Similarly, we found the highest proportion of unmet needs in rehabilitation and social and family support, whereas needs for medical and nursing care, personal care, and environmental needs were mostly met. The lack of community-based rehabilitation services has been underpinned by qualitative research, and studies have demonstrated inadequate community-based rehabilitation services and personal and family support for persons with neurological conditions (32) and brain injury (33).

The NPCS multiple regression model showed that residing in less central regions increased unmet needs at the 6-month follow-up. This result is in line with an Australian survey of rehabilitation services, which reported that more services were delivered in metropolitan areas compared with rural areas (14). However, our results are in contrast with the Canadian study on 435 multiple trauma survivors from 10 different trauma centres (15), which found no regional differences in perceived rehabilitation needs and barriers to rehabilitation services. One of the values of the Canadian healthcare system is the universal coverage and provision of services based on the needs of the population. Similarly, the healthcare system in Norway is also publicly financed and aims to provide universally accessible healthcare services, including rehabilitation. In general, unmet needs for medical care in Norway in 2019 were at the lowest rate among Nordic countries (< 1%), and approximately half the EU average, with waiting times as the main reason for unmet needs (34). Nevertheless, the reported unmet needs represent a substantial challenge for patients and health and rehabilitation services.

More profound overall injury and severe head injuries (AIS ≥ 3) were related to unmet needs at the 6-month follow-up. This is in line with a recently published study on factors associated with unmet needs in the moderate-to-severe TBI population in Europe, indicating that the probability of receiving rehabilitation depends primarily on injury-related factors (13). Discharge to specialized rehabilitation was also significantly related to unmet needs for community-based services in our study, and this factor could be interpreted as an indicator of injury severity (13). Patients with severe injuries receive a higher amount of healthcare and rehabilitation services than those with moderate injuries, thus we could expect that their needs would be substantially covered. However, we found that needs for community-based healthcare services, social support, and rehabilitation to a larger extent were unmet for this group. An explanation for this could be that patients who received subacute rehabilitation are a lower priority for community service providers in some municipalities. Additionally, ambulatory rehabilitation teams from the specialist healthcare service are few, hampering cooperation between rehabilitation levels. Information on available services and how patients could navigate the healthcare system may be insufficient. Patients may also have needed less therapy in the first 6 months than was estimated on discharge.

When analysing independent factors associated with the principal domains of NPCS, a similar pattern of predictors revealed that certain factors are common and consistently important. In the social support domain, having spinal injury AIS ≥ 3 or a pre-injury mental health or drug/alcohol condition increased the odds of unmet needs. The literature on spinal cord injuries indicates that the long-term care needed is higher than the care received, particularly related to psychosocial needs (35). Mental health or drug/alcohol conditions can affect social behaviour and thus negatively influence perceptions of social support. As expected, in the regression analyses of the NPCS rehabilitation subscale, injury severity, including having sustained profound injuries, severe head injury, and higher numbers of injuries, was associated with unmet rehabilitation needs.

### Strength and limitations

Strengths of this study include its prospective, multicentre design, large sample size across all ages, use of verified injury severity scores through the hospitals’ trauma registries, and a high 6-month follow-up rate. Overall, these strengths contribute to the robustness of the findings.

Concerning limitations, selection bias must be considered, as marginal groups may have lower inclusion and follow-up rates. Further, the study was conducted during the COVID-19 pandemic, and external factors might have influenced both the inclusion of the study participants and the services they received during this period. Moreover, patient-reported levels of services provided may be prone to recall bias.

In terms of assessing needs, the methods used for the estimation of rehabilitation needs have not previously been extensively validated in a trauma population or a Scandinavian healthcare setting and should be a focus for future research. Further, unmet needs for subacute rehabilitation may be underestimated, as the RCS E–Trauma does not measure delays in transfer to inpatient rehabilitation, the quality of the multidisciplinary treatment, or discharge plans. In addition, the organization of community-based services among municipalities was not assessed in this study.

### Conclusion

The inadequacy in service provision contributes to a high prevalence of unmet needs among patients during the initial 6 months post-injury. The finding calls for a more comprehensive evaluation of patients’ impairments and corresponding rehabilitation needs to support individuals recovering from traumatic injuries. The association between patient outcomes and service needs will be presented in future studies from this project. Projects evaluating longer-term rehabilitation needs after injury are also warranted.

## Supplementary Material

UNMET REHABILITATION NEEDS IN THE FIRST 6 MONTHS POST-INJURY IN A TRAUMA CENTRE POPULATION WITH MODERATE-TO-SEVERE TRAUMATIC INJURIES
