# Supplementary material for: UNMET REHABILITATION NEEDS IN THE FIRST 6 MONTHS POST-INJURY IN A TRAUMA CENTRE POPULATION WITH MODERATE-TO-SEVERE TRAUMATIC INJURIES
Source: J Rehabil Med. 2024 May 27;56:40078. doi: 10.2340/jrm.v56.40078 (PMC11151492; doi:10.2340/jrm.v56.40078)
Supplement: UNMET REHABILITATION NEEDS IN THE FIRST 6 MONTHS POST-INJURY IN A TRAUMA CENTRE POPULATION WITH MODERATE-TO-SEVERE TRAUMATIC INJURIES [file JRM-56-40078-s1.pdf]

Supplementary material has been published as submitted. It has not been copyedited, or typeset by Journal of Rehabilitation Medicine

Table SI. Factors associated with unmet needs at 6 months post-injury as assessed with the Needs and Provision Complexity Scale for the health and personal care domain in multiple logistic regression analysis.

Variables	OR (95% CI)	*p*-value
Sex, Female vs male[a]	1.321 (0.834–2.093)	0.236
Age (years)	1.009 (0.998–1.021)	0.118
Living with others vs. living alone[a]	1.080 (0.698–1.670)	0.729
Less central vs. central[a]	1.766 (1.176–2.651)	0.006
Pre-injury comorbidity (ASA), systemic disease vs. healthy[a]	1.014 (0.620–1.660)	0.956
Pre-injury mental health or drug/alcohol condition, no vs yes[a]	0.598 (0.347–1.031)	0.064
NISS ≥ 26 vs ≤ 25[a]	1.490 (0.891–2.490)	0.128
Number of injuries	0.950 (0.892–1.011)	0.108
Head AIS ≥ 3 vs < 3[a]	2.532 (1.471–4.358)	<0.001
Thorax AIS ≥ 3 vs <3 [a]	1.070 (0.649–1.766)	0.791
Abdomen AIS ≥ 3 vs < 3[a]	0.791 (0.414–1.513)	0.479
Spine AIS ≥ 3 vs < 3[a]	0.940 (0.524–1.688)	0.837
Lower Extremities AIS ≥ 3 vs < 3[a]	1.230 (0.688–2.201)	0.485
Discharge destination, specialized rehabilitation vs local hospital/home/other[a]	1.790 (1.051–3.049)	0.032

[a]Reference group

OR > 1 increases the odds of having a high level of unmet needs; OR < 1 decreases the odds of having a high level of unmet needs.

Model fit statistics: Hosmer and Lemeshow goodness-of-fit test $X^2 = 4.058$, df = 8, p = 0.168. Log likelihood = 585.640; Cox Snell $R^2 = 0.126$, Nagelkerke $R^2 = 0.168$.

AIS = Abbreviated Injury Scale; ASA = American Society of Anesthesiologists classification; CI = Confidence interval; NCI = Norwegian Centrality Index; NISS = New Injury Severity Score; OR = Odds ratio.

Table SII. Factors associated with unmet needs at 6 months post-injury as assessed with the Needs and Provision Complexity Scale for the social care and support needs domain in multiple logistic regression analysis.

Variables	OR (95% CI)	p-value
Sex, Female vs male[a]	1.365 (0.858–2.170)	0.189
Age (years)	1.001 (0.990–1.013)	0.801
Living with others vs. living alone[a]	1.290 (0.828–2.009)	0.260
Less central vs. central[a]	2.144 (1.422–3.234)	<0.001
Pre-injury comorbidity (ASA), systemic disease vs. healthy[a]	1.107 (0.672–1.826)	0.689
Pre-injury mental health or drug/alcohol condition, no vs yes[a]	0.465 (0.267–0.809)	0.007
NISS ≥ 26 vs ≤ 25[a]	1.363 (0.812–2.288)	0.241
Number of injuries	1.018 (0.954–1.085)	0.593
Head AIS ≥ 3 vs < 3[a]	1.005 (0.576–1.751)	0.987
Thorax AIS ≥ 3 vs < 3[a]	0.636 (0.380–1.063)	0.084
Abdomen AIS ≥ 3 vs < 3[a]	1.004 (0.521–1.935)	0.991
Spine ≥ 3 vs < 3[a]	1.813 (0.990–3.320)	0.054
Lower Extremities AIS ≥ 3 vs < 3[a]	2.922 (1.592–5.364)	<0.001
Discharge destination, specialized rehabilitation vs local hospital/home/other[a]	2.308 (1.350–3.945)	0.002

[a]Reference group

OR > 1 increases the odds of having a high level of unmet needs; OR < 1 decreases the odds of having a high level of unmet needs.

Model fit statistics: Hosmer and Lemeshow goodness-of-fit test $X^2 = 5.811$, df = 8, p = 0.668. Log likelihood = 577.465; Cox Snell $R^2 = 0.139$, Nagelkerke $R^2 = 0.185$.

AIS = Abbreviated Injury Scale; ASA = American Society of Anesthesiologists classification; CI = Confidence interval; NCI = Norwegian Centrality Index; NISS = New Injury Severity Score; OR = Odds ratio.

Table SIII. Factors associated with unmet needs at 6 months post-injury as assessed with the Needs and Provision Complexity Scale for the rehabilitation subscale in multiple logistic regression analysis.

Variables	OR (95% CI)	*p*-value
Sex, Female vs male[a]	1.216 (0.767–1.926)	0.405
Age (years)	1.004 (0.992–1.015)	0.524
Living with others vs. living alone[a]	0.936 (0.606–1.447)	0.767
Less central vs. central[a]	2.072 (1.378–3.115)	<0.001
Pre-injury comorbidity (ASA), systemic disease vs. healthy[a]	0.997 (0.610–1.631)	0.992
Pre-injury mental health or drug/alcohol condition, no vs yes[a]	0.465 (0.267–0.809)	0.007
NISS ≥ 26 vs ≤ 25[a]	1.669 (0.996–2.795)	0.052
Number of injuries	0.930 (0.873–0.992)	0.027
Head AIS ≥ 3 vs < 3[a]	2.557 (1.479–4.422)	<0.001
Thorax AIS ≥ 3 vs < 3[a]	1.081 (0.653–1.789)	0.762
Abdomen AIS ≥ 3 vs < 3[a]	0.545 (0.282–1.054)	0.071
Spine AIS ≥ 3 vs <3[a]	1.342 (0.746–2.412)	0.326
Lower Extremities AIS ≥ 3 vs < 3[a]	0.601 (0.348–1.039)	0.068
Discharge destination, specialized rehabilitation vs local hospital/home/other[a]	1.211 (0.716–2.048)	0.475

[a]Reference group

OR > 1 increases the odds of having a high level of unmet needs; OR < 1 decreases the odds of having a high level of unmet needs.

Model fit statistics: Hosmer and Lemeshow Goodness-of-fit test $X^2 = 7.298$, df = 8, p = 0.505. Log likelihood = 586.011; Cox Snell $R^2 = 0.123$, Nagelkerke $R^2 = 0.164$.

AIS = Abbreviated Injury Scale; ASA = American Society of Anesthesiologists classification; CI = Confidence interval; NCI = Norwegian Centrality Index; NISS = New Injury Severity Score; OR = Odds ratio.
